# In Vivo Antioxidant Effects Evaluation of Genetically Modified Astaxanthin-Rich Maize

**DOI:** 10.3390/foods15193384

**Published:** 2026-09-23

**Authors:** Xiyao Liu, Rui Wang, Jiaxi Lu, Yichun Hu, Min Li, Jing Chen, Deqian Mao, Lichen Yang

**Affiliations:** Key Laboratory of Public Nutrition and Health, National Health Commission of the People’s Republic of China, National Institute for Nutrition and Health, Chinese Center for Disease Control and Prevention, No. 29 Nanwei Road, Xicheng District, Beijing 100050, China; liuxiyao1997@163.com (X.L.); wangrui@ninh.chinacdc.cn (R.W.); lujx@ninh.chinacdc.cn (J.L.); huyc@ninh.chinacdc.cn (Y.H.); limin@ninh.chinacdc.cn (M.L.); chenjing@ninh.chinacdc.cn (J.C.)

**Keywords:** rats, oxidative stress, astaxanthin, maize, nutritional components, feeding experiment

## Abstract

Astaxanthin-rich maize has been developed through genetic modification as a potential dietary source of astaxanthin. This study aimed to evaluate the nutritional components and the antioxidant effects of astaxanthin-rich maize. The antioxidant effects were assessed from a 60-day feeding experiment with 9-month-old male SD rats. The results showed that the content of astaxanthin in astaxanthin-rich maize was 24.91 μg/g. Compared with rats fed conventional feed or parental maize, those fed astaxanthin-rich maize showed higher serum superoxide dismutase (SOD) and glutathione (GSH) (*p* < 0.05). Serum malondialdehyde (MDA) in the astaxanthin-rich maize group was lower than that in the conventional feed group (*p* < 0.05). Liver protein carbonyl and MDA in the astaxanthin-rich maize group were lower than those in the conventional feed group (*p* < 0.05). Liver SOD activity, glutathione peroxidase (GPX) and GSH level in the astaxanthin-rich maize groups were higher than those in the conventional feed group or the parental maize group (*p* < 0.05). The astaxanthin-rich maize showed positive antioxidant effects in several indicators. In the future, antioxidant evaluations of the astaxanthin-rich maize need to be conducted over a broader concentration range and using more oxidative stress indicators.

## 1. Introduction

Astaxanthin is a xanthophyll carotenoid ubiquitous in nature. It is present at high levels in various organisms, such as shrimp, crab, salmon and algae [1]. The conjugated polyene chain, and the unsaturated keto and hydroxyl groups in the astaxanthin molecule contribute to its strong antioxidant properties. It can scavenge free radicals and reactive oxygen species (ROS), thereby helping to prevent chronic noninfectious diseases such as cardiovascular disease, diabetes, and tumors [2]. Astaxanthin cannot be synthesized by the human body [3]. Toxicological studies have shown that natural astaxanthin has no adverse effects on humans or animals [4,5]. However, excessive intake of it may cause pigmentation. Therefore, given its health benefits and market prospects, the long-term safety of astaxanthin intake at various doses requires further investigation.

With the development of transgenic technology, significant progress has also been made in the cultivation of new transgenic crops. Transgenic technology also provides a new way to efficiently produce natural astaxanthin with high bioactivity at low cost [6,7]. Previous studies have already increased the content of astaxanthin in plants through transgenic technology and conducted functional evaluations. A study increased the astaxanthin content in the leaves of Arabidopsis thaliana by overexpressing the Chlamydomonas reinhardtii β-carotene ketolase (CrBKT) in this species. The results showed that the oxidative stress tolerance of the plants was enhanced [8]. Another study increased the astaxanthin content in rice endosperm by introducing four synthetic genes (*sZmPSY1*, *sPaCrtI*, *sCrBKT*, and *sHpBHY*). The assessment also revealed that the antioxidant capacity of genetically modified rice was significantly higher than that of the wild type [9].

As an important food crop, maize is one of the earliest genetically modified crops to be commercialized on a large scale [10]. Astaxanthin-rich maize has also been produced in previous studies [11,12]. Among them, the research team from the Biotechnology Research Institute of Chinese Academy of Agricultural Sciences increased lycopene synthesis and redirected lycopene flux. Two constructs, pBDEN-CP-BZ and pBDEN-CP-BZ-LCYERNAi, were formed. The *Agrobacterium*-mediated transformation was employed to introduce the constructs into Hi-II maize. RNAi was used to reduce the conversion of lycopene to α-carotene and to promote its conversion into the β-carotene pathway. Then various combinations of β-carotene ketolase and β-carotene hydroxylase genes were overexpressed in seed. The seed-specific bidirectional promoter was combined with these genes to form different expression constructs and to drive the conversion from carotenoids to astaxanthin in maize [12]. The maize obtained through backcross breeding also showed a high content of astaxanthin. The astaxanthin content in this kind of maize was significantly increased and remained stable during storage (4 °C). Although transgenic technology could enhance the nutritional content of astaxanthin, its safety is still under public debate. Potential risks, such as changes in nutritional components and adverse effects, need to be evaluated.

Overall, most previous studies have mainly focused on the cultivation of transgenic astaxanthin-rich plants and conducted in planta antioxidant properties evaluations. However, comprehensive assessments of nutritional components and in vivo antioxidant properties remain limited. Therefore, the aims of this study were (1) to evaluate the nutritional components of astaxanthin-rich maize produced by the Biotechnology Research Institute of Chinese Academy of Agricultural Sciences; (2) to explore the effect of astaxanthin-rich maize on antioxidant effects through rat feeding experiments.

## 2. Materials and Methods

### 2.1. Materials

Astaxanthin-rich maize and the parental control maize (Zhengdan958 maize) were both provided by the Biotechnology Research Institute, Chinese Academy of Agricultural Sciences. The astaxanthin-rich maize in this study was developed using the same genetic constructs and breeding strategy as described in the study of this Institute, with no additional genetic modifications [12]. The maize samples used for the nutritional evaluation and those employed in the experiment were all from the same batch. They were grown in the same experimental field located in Langfang, Hebei Province, China. Planting, irrigation, and pest control were all managed uniformly by the Institute. The maize samples were harvested at the maturity stage in the same season under dry weather conditions in 2020. After harvest, the maize kernels were dried, ground, and stored under identical conditions until use. For each group of maize (astaxanthin-rich maize and parental maize), 500 g of kernels were randomly selected and mixed thoroughly for analysis of nutritional components and astaxanthin content.

### 2.2. Nutrients Components Analysis

The contents of main nutrients, minerals and vitamins in astaxanthin-rich maize and parental maize were determined by the Beijing Institute of Nutrition Sources in China (National Laboratory Certification CNAS No. L2678, Measurement Certificate No. 2004010329Z) according to Chinese national standard methods. The results were then compared with the reference ranges of the Organisation for Economic Cooperation and Development (OECD, 2002) [13] and the International Life Sciences Institute (ILSI, version 7.0, 2019) [14]. Three parallel samples were analyzed for all nutrient determinations. The specific detection methods are as follows.

#### 2.2.1. Determination of Main Nutrients

The protein, fat, ash, water, and dietary fiber content were determined using the Kjeldahl method [15], acid hydrolysis [16], muffle furnace combustion [17], oven-drying method [18], and enzymatic–gravimetric method [19], respectively. The carbohydrate content was calculated by subtracting the contents of water, protein, fat, ash, and dietary fiber from the total sample weight. The detailed method description can be found in the Supplementary Material.

#### 2.2.2. Determination of Minerals

The calcium, potassium, phosphorus, magnesium, manganese, iron, copper, zinc, and sodium in the maize samples were determined by Inductively Coupled Plasma–Optical Emission Spectrometry (ICP-OES) [20]. The selenium in the maize samples was determined by Hydride Generation Atomic Fluorescence Spectrometry [21]. The limits of quantitation and precision of all elements met the requirements of national standards [20,21]. The detailed method description can be found in the Supplementary Material.

#### 2.2.3. Determination of Vitamins

Vitamin A, δ-tocopherol, γ-tocopherol, β-tocopherol, and α-tocopherol in the maize samples were determined by reversed-phase high-performance liquid chromatography [22]. Vitamin K1 was determined by high-performance liquid chromatography–fluorescence detection [23]. β-carotene, vitamin B1 and B2 were determined by high-performance liquid chromatography [24,25,26]. The contents of niacin [27], folate [28] and biotin [29] in maize was determined by microbiological assay after acid hydrolysis with sulfuric acid solution. The detailed method description can be found in the Supplementary Material.

#### 2.2.4. Determination of Astaxanthin

Astaxanthin content was determined using high-performance liquid chromatography (HPLC) by the Institute of Animal Science, Chinese Academy of Agricultural Sciences, according to Chinese national standard method [30]. The sample was analyzed after grinding and homogenization. Chromatographic separation was carried out on C30 column (4.6 × 250 mm, 5 μm) with mobile phase A consisting of methanol–methyl tert-butyl ether (92%) and mobile phase B consisting of ultrapure water (8%) at a flow rate of 1.0 mL/min. The injection volume was 10 μL. The column temperature was maintained at 25 °C, and detection was carried out at 474 nm. Quantification was achieved using an external standard method [30].

### 2.3. Antioxidant Effects Assessment Methods

#### 2.3.1. Grouping and Dosage Information

Healthy 9-month-old male SD rats were employed in this study. The use of male rats avoided potential confounding effects from estrogen, which possesses inherent antioxidant properties. The rats of SPF grade [Beijing Vital River Laboratory Animal Technology Co., Ltd., license: SCXK (Beijing, China), 2016-0006] were raised in the Institute of Laboratory Animals Science, Chinese Academy of Medical Sciences & Peking Union Medical College [license: SYXK Beijing, China, 2018-0019], with an ambient temperature of 20–23 °C, humidity of 40–70%, artificial lighting/dark cycle for 12 h, and ventilation ≥ 15 times/hour. The rats were kept under ad libitum conditions during the experiment. All feeding operations during the experiment met the requirements of animal welfare. All methods and results are reported in accordance with ARRIVE guidelines for animal experiments. Ethical approval for this experiment was obtained from the ethics committee of the National Institute for Nutrition and Health, China Center for Disease Control and Prevention (No. 2020-NINH-IACUC-034).

The experiment was carried out from October to December 2020. After 7 days of adaptive feeding, the rats were randomly divided into 4 groups. Feed for each group is shown in Table 1. The feed was formulated based on the nutrient requirements in the AIN-93M formula for adult rodents maintenance published by the American Nutrition Institute [31]. For the astaxanthin-rich maize groups and the parental maize (PM) group, the maximum maize incorporation ratio was determined by the protein requirement. The diet was designed to contain 10% protein. The remaining protein deficiency in the maize groups was made up with casein, which is of high quality and good palatability [31]. By calculation, the feed of PM group contained 82.5% parental maize. The feed of high-dose astaxanthin-rich maize group (HDAM) contained 83% astaxanthin-rich maize. In this way, the feed could be formulated in a balanced and appropriate manner to meet the protein requirement and the normal growth needs of the animals. The group containing 50% of the astaxanthin dose of the HDAM group was defined as the low-dose astaxanthin-rich maize (LDAM) group. Other nutrients were formulated according to AIN-93M formula, after deducting the nutrient contents contributed by maize [31]. The prepared feed was dried and stored in vacuum package for experimental use. To establish a broader range of astaxanthin dose gradients, three additional groups of rats were administered astaxanthin via gavage (Table S1). The details are described in the Supplementary Materials.

#### 2.3.2. Indicators and Laboratory Analysis

The experiment lasted for 60 days. The animal activity, fur, food intake, excretion, toxicity signs, and death were observed daily. The food intake and body weight of the rats were recorded every week. The records were made on the same weekday morning every week. After the experiment, the rats were killed after a 12 h fast for blood collection and liver removal. The rats were euthanized by exsanguinating after anesthesia. The blood was centrifuged to separate the serum within 2 h after collection (at 4 °C, 2500 rpm, for 10 min). The serum was frozen at −80 °C for preservation. The liver was removed and placed in an ice-cooled Petri dish. Then, the blood and other residues were washed with PBS solution. The liver was weighed and then crushed in a frozen state. The animal experimental oxidative stress indicators included in the antioxidant function evaluation standard for health-care food in China were detected using commercial kits (Nanjing Jiancheng Bioengineering Institute, Nanjing, China). These included: (1) lipid oxidation products: malondialdehyde (MDA) and 8-isoprostane; (2) protein oxidation products: protein carbonyl; (3) antioxidant enzymes: superoxide dismutase (SOD) and glutathione peroxidase (GPX); (4) antioxidant: glutathione (GSH) [32]. According to the instructions of the kits, the levels of GPX, MDA, SOD, GSH and protein carbonyl were tested using spectrophotometry, and 8-isoprostane was detected by enzyme-linked immunosorbent assay (ELISA) [32]. The first two categories were oxidative stress products, and a significant decrease indicated positive antioxidant function. The last two categories were antioxidant-related substances, and a significant increase indicates positive antioxidant function.

### 2.4. Statistical Analyses

Data analyses were performed using SPSS software, version 25.0 (IBM Corp., Armonk, NY, USA). Continuous variables were presented as Mean ± standard deviation (SD). In the assessment of body weight and food intake, repeated-measures analysis of variance was used to test the changes. The effects of time and the interaction between group and time were analyzed. Mauchly’s test was used to verify the sphericity assumption. When the spherical assumption was rejected, the Greenhouse–Geisser method was used for correction. The intake data were normalized based on body weight. Analysis of variance was used to compare the differences in oxidative stress among the groups. Post hoc comparisons were performed using Tukey’s test for variables with homogeneous variances, whereas Games–Howell test was used for variables with heterogeneous variances. The astaxanthin-rich maize groups were compared with the conventional feed group as well as the parental maize group. Statistical comparisons involving the gavage groups are detailed in the Supplementary Materials. Tukey’s test was used for post hoc comparisons. A two-sided *p* < 0.05 was considered statistically significant.

## 3. Results

### 3.1. Nutritional Components of Astaxanthin-Rich Maize

The content of astaxanthin in astaxanthin-rich maize was 24.91 μg/g dry weight (DW), while no astaxanthin was detected in parental maize. Table 2, Table 3 and Table 4 show the content of main nutrients, minerals and vitamins in parental maize and astaxanthin-rich maize. The content of β-tocopherol in astaxanthin-rich maize was higher than that in parental maize. The contents of other nutrients were similar between the two groups of maize. The nutritional components of astaxanthin-rich maize were compared with reference ranges of OECD and ILSI. The contents of carbohydrate, selenium, vitamin B1, and vitamin B2 were below OECD or ILSI range.

### 3.2. Antioxidant Effects Assessment

#### 3.2.1. Body Weight and Diet of Rats

During the experiment, the rats had normal eating, drinking and activity, and no abnormal reactions or toxic symptoms were observed.

Table 5 shows that the change in body weight was statistically significant, but the interaction between the groups and the measurement time was not significant (*p* > 0.05). The body weights of rats in each group are shown in Table S2. No statistically significant differences in body weight were observed between the two astaxanthin-rich maize groups (HDAM and LDAM) and the CF or PM groups throughout the 8-week experimental period (*p* > 0.05). Table 5 also shows that the change in food intake was statistically significant, and the interaction between the groups and the measurement time was also significant (*p* < 0.05). As shown in Table S3, the HDAM group exhibited significantly different food intake from the CF group at weeks 2, 6, and 8 (*p* < 0.05) but not from the PM group (*p* > 0.05). As a supplemental analysis, the gavage groups were analyzed together with the CF group, and the changes in body weight and food intake, as well as the interaction between the groups and the measurement time, were statistically significant; see Tables S4–S6 for detailed data.

#### 3.2.2. Oxidative Stress Indicators

Table 6 shows the serum oxidative stress indicators and the differences among groups. The SOD activity in the HDAM and LDAM groups was higher than that in the PM group or the CF group (*p* < 0.05). The MDA concentration in the HDAM group was significantly lower than that in the CF group, while that in the HDAM and LDAM group was higher than that in the PM group (*p* < 0.05). GSH level in the HDAM group and the LDAM group was significantly higher than that in the CF group (*p* < 0.05). The statistical comparisons of serum indicators between the three gavage groups and the CF group are presented in Table S7. In the gavage groups, serum SOD and GSH levels were significantly higher than those in the CF group, while MDA level was significantly lower.

Table 7 shows the results of liver oxidative stress indicators. The protein carbonyl concentration in the HDAM group was lower than that in the CF group (*p* < 0.05). The SOD activity in the LDAM group was higher than that in the CF group and PM group (*p* < 0.05). The MDA concentration in the HDAM group was significantly lower than that in the CF group. The GSH level in the LDAM group was higher than that in the CF group and the PM group (*p* < 0.05). The GPX activity of rats in the two astaxanthin-rich maize groups was lower than that in the CF group but higher than that in the PM group (*p* < 0.05). Compared with the HDAM group, the LDAM group showed significantly higher levels of SOD, MDA, and GSH (*p* < 0.05). Compared with the CF group, the gavage groups showed significantly lower liver protein carbonyl, which is shown in Table S8.

## 4. Discussion

Nutrition assessment is an important part of safety evaluation of genetically modified food [33]. According to the substantial equivalence principle, the main nutrients, minerals and vitamins in astaxanthin-rich maize were determined and compared with those in parental maize. Most nutrients in astaxanthin-rich maize were within the reference ranges of the OECD or ILSI and were generally similar to those in parental maize. The contents of carbohydrate, selenium, vitamin B1, and vitamin B2 were below the reference ranges of OECD or ILSI. This may be attributed to differences in genetic background, environmental growth conditions (light, temperature, water), and planting management. Overall, the levels of nutritional components of maize in this study were similar to the reference ranges.

The β-tocopherol content in astaxanthin-rich maize is higher than that in parental maize. The possible reason is that genetic modification alters the metabolic pathways. Astaxanthin and β-tocopherol are both liposoluble antioxidants. Genetic modification may regulate the key enzyme genes of β-tocopherol. However, this explanation is speculative, and no direct research evidence has been found. These results indicate that the introduction of the astaxanthin trait had no adverse effects on the existing nutrient profile. According to Codex Alimentarius (CAC/GL 45-2003) [34], the differences in nutritional components are not safety issues, and they can be used to inform subsequent evaluations. We consider that these differences do not exceed safety assessment thresholds.

In this study, no astaxanthin was detected in parental maize, while the content of astaxanthin in astaxanthin-rich maize was 24.91 μg/g DW. Previous studies have reported that astaxanthin has been biosynthesized in various plants via genetic modification. The content of astaxanthin in transgenic tomato fruit [35], rice endosperm [9], and maize [6] is 16.1 mg/g DW, 9.8–16.2 μg/g DW, and 10.8–16.8 μg/g DW, respectively. Our maize had higher astaxanthin than rice and previous maize but lower than tomato, which has abundant precursors [9]. Different types of plants have different genetic backgrounds for the production of carotenoids, and the construction methods of genetic modification are also different [6,9,35]. In addition, the astaxanthin content of maize in this study was lower than the value 47.76–111.82 μg/g DW reported by the Biotechnology Research Institute of Chinese Academy of Agricultural Sciences [12], which may be attributed to different planting conditions, growing environment, and detection methods. Environmental conditions during crop growth affect carotenoid content [36,37]. Therefore, we suggest that climate conditions, such as moisture and light, as well as soil type and seed density, may cause variations in the astaxanthin content among different batches of maize.

In the 60-day feeding experiment, the food intake and activity of rats were observed to be normal. Feeding astaxanthin-rich maize had no adverse effects on body weight or food intake. Compared with the parental maize group or the conventional feed group, serum antioxidant enzymes, serum antioxidants, serum lipid oxidation products, liver antioxidant enzymes, liver lipid oxidation products, liver antioxidants and liver protein oxidation products in the astaxanthin-rich maize groups showed positive results. It can be considered that astaxanthin-rich maize has antioxidant effects. However, the levels of some oxidative stress indicators did not achieve the expected results. And it could not be concluded that the HDAM group exhibited stronger antioxidant effects than the LDAM group. In the three additional groups of rats receiving astaxanthin via gavage, oxidative stress-related indicators and the dose–response relationship were analyzed. The results show that although the astaxanthin gavage groups exhibited significant differences from the CF group in several serum and liver indicators, no clear dose-dependent trend was observed across the three astaxanthin dose levels. These findings suggest that the effects of astaxanthin on oxidative stress indicators may follow a non-linear pattern under the current experimental conditions, and formal trend analyses are warranted in future studies.

Previous human and animal studies have reported the antioxidant effects of astaxanthin [38,39,40,41,42]. For example, in an animal experiment, mice were fed with a conventional diet containing 0.01% astaxanthin for 6 weeks. Liver weight, oxidative stress-related enzyme activities, and mRNA expression were measured. It was shown that the levels of SOD, catalase and GPX in the intervention group significantly increased, while the level of MDA significantly decreased [41]. Another study used db/db mice to establish a type 2 diabetes model. The intervention group was fed a diet containing 0.02% astaxanthin. After 12 weeks, the results showed that astaxanthin could significantly reduce the level of 8-OHdG [43]. Astaxanthin can reduce the levels of reactive oxygen species and reactive nitrogen species, reduce protein oxidation products, increase the activity of antioxidant enzymes, protect mitochondrial function and structural integrity, and thus delay the progression of many diseases [44,45,46]. From the perspective of molecular mechanisms, astaxanthin in the body can activate nuclear factor erythroid 2-related factor 2 (Nrf2) by promoting its release from Kelch-like ECH-associated protein 1 (Keap1) or Kelch-like ECH-associated protein 2 (Keap2). After release, Nrf2 enters the cell nucleus and binds to the antioxidant response element (ARE), activating antioxidant enzymes such as SOD and GPX, thereby overall enhancing the antioxidant level [47,48]. The inhibition of lipid peroxidation and protein oxidation by astaxanthin may be due to the fact that astaxanthin can modify the properties of cell membranes, stabilize membrane structure and eliminate free radicals [49].

Maize is a suitable plant for the efficient production of astaxanthin [6,12]. However, the literature evaluating the food safety and function of astaxanthin-rich maize produced by transgenic technology is limited. Previous studies mainly evaluated the absorption and bioavailability of astaxanthin from the perspective of animal production. For example, feeding experiments showed that astaxanthin could accumulate in the yolk after laying hens were fed astaxanthin-rich maize, which did not affect the production and quality of eggs. The serum SOD concentration of hens in the astaxanthin-rich maize group significantly increased, while the MDA concentration decreased [12,50]. We suggest that astaxanthin, as a nutrient, when existing in food, could participate in food matrix effects. Various nutrients, phytochemicals, and other complex components have a natural regulatory effect on digestion and absorption [51,52]. However, supplementing with a single nutrient may have adverse effects on this regulatory function. From this perspective, astaxanthin intake from natural food sources could be considered superior to commercial astaxanthin supplements. Future research should further focus on the underlying pharmacokinetic mechanism.

This study provides regulatory agencies with foundational data on the nutritional equivalence and preliminary antioxidant activity of astaxanthin-rich maize, serving as a reference for its safety assessment and approval. It also offers a basis for the development of genetically modified crops and other bioactivity evaluation studies. Future research on the antioxidant effects of astaxanthin can build upon the experimental group and dosage design of this study to conduct more in-depth explorations. People who need to enhance their antioxidant capacity can also benefit from it, thereby preventing diseases and promoting health. However, limitations of this study should also be acknowledged. First, this study did not identify any pattern of dose–response. The astaxanthin content in the astaxanthin-rich maize used in this study was limited, which may affect the exploration of the dose-dependent relationship. This limitation is a critical point, as it directly affects the translational potential of astaxanthin-rich maize. Future research should aim to increase the content of astaxanthin in maize, which would allow for a more comprehensive assessment of its dose-dependent efficacy. Second, the assessment of antioxidant effects is based on several classic indicators, without conducting more in-depth exploration of other mechanism indicators. Third, we did not systematically evaluate the tolerance to gavage and did not set up a separate control group for gavage administration, which may affect the analysis of the antioxidant results in these supplementary gavage groups. However, through observation, we did not find any significant adverse effects of gavage on the life and activities of the rats.

## 5. Conclusions

The nutritional components of the astaxanthin-rich maize detected in this study showed substantial equivalence to those of parental maize. No adverse effects on the life and normal activities of rats were found. In this study, some of the antioxidant indicators in the astaxanthin-rich maize groups showed positive effects. It can be considered that astaxanthin-rich maize has a certain antioxidant effect. However, the results of some indicators in this study did not meet expectations, and no dose–response relationship was found. It is necessary to consider further increasing the content of astaxanthin in the genetically modified maize and to design a more appropriate dose gradient for dose–response exploration. Furthermore, future research can also explore more indicators and focus on the underlying pathways involved in the antioxidant effects of astaxanthin-rich maize.

## Figures and Tables

**Table 1:** Grouping and feeding of rats.

Group	Treatment Group	Sample Size	Feeding	Astaxanthin Content (μg/g)
Control groups	Conventional feed (CF) group	10	AIN-93M ordinary diet	0
	Parental maize (PM) group	10	Formulated diet containing 82.5% parental maize	0
Experimental groups	High-dose astaxanthin-rich maize (HDAM) group	16	Formulated diet containing 83% astaxanthin-rich maize	20.68
	Low-dose astaxanthin-rich maize (LDAM) group	10	Formulated diet containing 41.5% astaxanthin-rich maize	10.34

**Table 2:** Content of main nutrients in astaxanthin-rich maize and parental maize (Mean ± SD) ^c^.

Nutrients ^a^	Astaxanthin-Rich Maize	Parental Maize	Reference Range ^b^	
			OECD	ILSI
Protein (g/100 g)	9.40 ± 0.01	9.04 ± 0.13	6.0–12.7	5.72–17.26 (10.20)
Fat (g/100 g)	4.2 ± 0.10	3.7 ± 0.10	3.1–5.8	1.37–7.83 (3.79)
Carbohydrate (g/100 g)	69.1 ± 0.18	70.8 ± 0.25	82.2–82.9	77.4–89.7 (84.6)
Ash content (g/100 g)	1.4 ± 0.003	1.4 ± 0.01	1.1–3.9	0.616–6.282 (1.395)
Water content (g/100 g)	8.29 ± 0.16	8.40 ± 0.12	7–23	5.1–40.7 (14.4)
Fiber (g/100 g)	7.65 ± 0.21	6.64 ± 0.12	/	5.78–35.31 (13.66)

^a^ Three parallel samples were included for the determination of all nutrients. Protein, fat, carbohydrate, fiber and ash content were measured on a dry weight basis. ^b^ OECD: Organisation for Economic Cooperation and Development; ILSI: the International Life Sciences Institute; the range is shown from the minimum to the maximum, with the average in parentheses. ^c^ SD: standard deviation.

**Table 3:** Content of minerals in astaxanthin-rich maize and parental maize (Mean ± SD) ^c^.

Nutrients ^a^	Astaxanthin-Rich Maize	Parental Maize	Reference Range ^b^	
			OECD	ILSI
Calcium (mg/kg)	38.5 ± 2.12	45.9 ± 2.54	30–150	11.8–1010 (42.1)
Potassium (g/kg)	3.94 ± 0.23	4.11 ± 0.21	3.2–7.2	1.810–6.030 (3.655)
Phosphorus (mg/100 g)	325 ± 14.13	327 ± 21.7	230–750	130–552 (312)
Magnesium (mg/kg)	1200 ± 100.22	1170 ± 75.85	820–10,000	594–1940 (1199.7)
Manganese (mg/kg)	4.75 ± 0.14	4.80 ± 0.26	/	1.69–14.3 (6.34)
Iron (mg/kg)	15.1 ± 0.52	15.6 ± 0.79	1–100	9.51–191 (20.11)
Copper (mg/kg)	1.27 ± 0.03	1.36 ± 0.04	0.9–10	0.55–21.2 (1.72)
Selenium (mg/kg)	0.012 ± 0.001	0.013 ± 0.001	0.01–1	0.03–1.51 (0.26)
Zinc (mg/kg)	21.7 ± 1.08	21.3 ± 0.79	12–30	6.5–42.6 (22.3)
Sodium (mg/kg)	<10	<10	0–1500	0.17–731.54 (21.54)

^a^ Three parallel samples were included for the determination of all nutrients. All data were measured on a dry weight basis. ^b^ OECD: Organisation for Economic Cooperation and Development; ILSI: the International Life Sciences Institute; the range is shown from the minimum to the maximum, with the average in parentheses. ^c^ SD: standard deviation.

**Table 4:** Content of vitamins in astaxanthin-rich maize and parental maize (Mean ± SD) ^c^.

Nutrients ^a^	Astaxanthin-Rich Maize	Parental Maize	Reference Range ^b^	
			OECD	ILSI
Vitamin A (μg/100 g)	<10	<10	0.049–0.218	0.019–8.020 (0.457)
Vitamin K_1_ (μg/100 g)	<1.5	<1.5	/	/
Vitamin B_1_ (mg/100 g)	0.225 ± 0.01	0.227 ± 0.01	0.23–0.86	0.126–4.000 (0.370)
Vitamin B_2_ (mg/100 g)	<0.05	<0.05	0.025–0.56	0.050–0.735 (0.196)
δ-tocopherol (mg/100 g)	0.218 ± 0.01	0.207 ± 0.01	/	0.038–1.461 (0.098)
γ-tocopherol (mg/100 g)	3.09 ± 0.09	2.95 ± 0.11	/	0.156–5.861 (1.814)
β-tocopherol (mg/100 g)	0.374 ± 0.03	<0.04	/	0.056–1.980 (0.106)
α-tocopherol (mg/100 g)	0.424 ± 0.02	0.211 ± 0.001	/	/
β-carotene (mg/100 g)	0.838 ± 0.19	0.604 ± 0.03	/	0.019–8.020 (2.716)
Niacin (mg/100 g)	1.5 ± 0.02	1.3 ± 0.02	0.93–7.0	0.742–4.694 (2.074)
Folate (mg/100 g)	0.137 ± 0.003	0.101 ± 0.001	/	0.009–0.350 (0.055)
Biotin (μg/100 g)	6.77 ± 0.07	8.16 ± 0.05	/	/

^a^ Three parallel samples were included for the determination of all nutrients. All data were measured on a dry weight basis. ^b^ OECD: Organisation for Economic Cooperation and Development; ILSI: the International Life Sciences Institute; the range is shown from the minimum to the maximum, with the average in parentheses. ^c^ SD: standard deviation.

**Table 5:** The results of the repeated-measures analysis of variance ^a^.

Variables	Effects	F	df	*p*	η^2^
Body weight	Time	152.416	1.413	<0.001	0.784
	Time×group	2.214	4.238	0.075	0.137
Food intake	Time	36.675	2.423	<0.001	0.647
	Time × group	3.001	7.270	0.010	0.310

^a^ Mauchly’s test indicated a violation of sphericity (*p* < 0.05). Greenhouse–Geisser corrected results are reported.

**Table 6:** Effect of astaxanthin-rich maize feeding experiment on serum oxidative stress indicators of rats (Mean ± SD) ^d^.

Group	CF Group (*n* = 10)	PM Group (*n* = 9)	HDAM Group (*n* = 16)	LDAM Group (*n* = 10)	F	df	*p*
Protein carbonyl (nmol/mL)	0.68 ± 0.22	0.57 ± 0.34	0.94 ± 0.44	0.97 ± 0.29	3.117	3	0.037
SOD (U/mL)	366.83 ± 26.16	378.18 ± 24.51	418.43 ± 39.97 ^ab^	412.75 ± 20.46 ^a^	8.189	3	<0.001
MDA (nmol/mL) ^c^	15.45 ± 4.06	5.55 ± 1.06	8.75 ± 1.75 ^ab^	13.53 ± 5.76 ^b^	28.671	3	<0.001
GSH (mg/L)	68.84 ± 13.35	104.23 ± 28.24	100.01 ± 21.77 ^a^	124.68 ± 27.91 ^a^	9.857	3	<0.001
GPX (U/mL)	925.94 ± 213.68	871.54 ± 328.68	853.48 ± 154.90	891.51 ± 276.76	0.215	3	0.886
8-isoprostane (ng/L)	63.80 ± 43.97	65.82 ± 53.38	66.83 ± 42.10	77.69 ± 46.25	0.435	3	0.729

^a^ Compared with the CF group, *p* < 0.05. ^b^ Compared with the PM group, *p* < 0.05. ^c^ Data for MDA were analyzed using Welch’s ANOVA due to heterogeneity of variances. ^d^ SD: standard deviation; SOD: superoxide dismutase; MDA: malondialdehyde; GSH: glutathione; GPX: glutathione peroxidase; CF: conventional feed; PM: parental maize; HDAM: high-dose astaxanthin-rich maize; LDAM: low-dose astaxanthin-rich maize.

**Table 7:** Effect of astaxanthin-rich maize feeding experiment on liver oxidative stress indicators of rats (Mean ± SD) ^e^.

Group	CF Group (*n* = 10)	PM Group (*n* = 9)	HDAM Group (*n* = 16)	LDAM Group (*n* = 10)	F	df	*p*
Protein carbonyl (nmol/mg prot) ^d^	10.24 ± 4.46	5.28 ± 1.76	3.47 ± 2.53 ^a^	6.35 ± 3.64	6.164	3	0.005
SOD (U/mg prot)	349.88 ± 44.83	308.55 ± 28.95	360.66 ± 73.11	536.54 ± 107.46 ^abc^	19.880	3	<0.001
MDA (nmol/mg prot)	1.07 ± 0.22	0.88 ± 0.18	0.72 ± 0.18 ^a^	0.94 ± 0.19 ^c^	7.118	3	0.001
GSH (mg/g prot)	1.49 ± 0.53	1.16 ± 0.41	1.43 ± 0.68	2.15 ± 0.29 ^abc^	6.260	3	0.001
GPX (U/mg prot)	539.18 ± 86.58	182.51 ± 64.25	304.29 ± 76.54 ^ab^	401.74 ± 123.88 ^ab^	27.757	3	<0.001
8-isoprostane (ng/g prot)	92.19 ± 17.57	81.24 ± 12.69	72.32 ± 29.84	68.04 ± 15.55	2.186	3	0.109

^a^ Compared with the CF group, *p* < 0.05. ^b^ Compared with the PM group, *p* < 0.05. ^c^ Compared with the HDAM group, *p* < 0.05. ^d^ Data for protein carbonyl were analyzed using Welch’s ANOVA due to heterogeneity of variances. ^e^ SD: standard deviation; SOD: superoxide dismutase; MDA: malondialdehyde; GSH: glutathione; GPX: glutathione peroxidase, CF: conventional feed; PM: parental maize; HDAM: high-dose astaxanthin-rich maize; LDAM: low-dose astaxanthin-rich maize.

## Data Availability

The data presented in this study are available on request from the corresponding authors. The data are not publicly available due to the collaborative research agreement and intellectual property restrictions.

## Supplementary Materials

The following supporting information can be downloaded at https://www.mdpi.com/article/10.3390/foods15193384/s1: Table S1: Grouping and feeding of rats; Table S2: Body weight changes and the comparison among astaxanthin-rich maize groups and control groups (g, Mean ± SD); Table S3: Food intake changes and the comparison among astaxanthin-rich maize groups and control groups (g/kg, Mean ± SD); Table S4: The results of the repeated-measures analysis of variance; Table S5: Body weight changes and the comparison among gavage groups and control group (g, Mean ± SD); Table S6: Food intake changes and the comparison among gavage groups and control group (g/kg, Mean ± SD); Table S7: Effect of astaxanthin-rich maize feeding experiment on serum oxidative stress indicators of rats (Mean ± SD); Table S8. Effect of astaxanthin-rich maize feeding experiment on liver oxidative stress indicators of rats (Mean ± SD).

## Abbreviations

The following abbreviations are used in this manuscript:

SD	Standard deviation
SOD	Superoxide dismutase
MDA	Malondialdehyde
GSH	Glutathione
GPX	Glutathione peroxidase
ROS	Reactive oxygen species
OECD	Organisation for Economic Cooperation and Development
ILSI	the International Life Sciences Institute
HPLC	High-performance liquid chromatography
DW	Dry weight
ELISA	Enzyme-linked immunosorbent assay
