# Supplementary material for: In Vivo Antioxidant Effects Evaluation of Genetically Modified Astaxanthin-Rich Maize
Source: Foods. 2026 Sep 23;15(19):3384. doi: 10.3390/foods15193384 (PMC13664148; doi:10.3390/foods15193384)
Supplement: Supplementary file 1 [file foods-15-03384-s001.zip › foods-4519059-supplementary.pdf]

Supplementary Materials

Nutritional components and in vivo antioxidant capacity evaluation of genetically modified astaxanthin-rich maize

Xiyao Liu, Rui Wang, Jiaxi Lu, Yichun Hu, Min Li, Jing Chen, Deqian Mao * and Lichen Yang *

Key Laboratory of Public Nutrition and Health, National Health Commission of the People's Republic of China, National Institute for Nutrition and Health, Chinese Center for Disease Control and Prevention, No.29 Nanwei Road, Xicheng District, Beijing 100050, China; liuxiyao1997@163.com (X.L.); wangrui@ninh.chinacdc.cn (R.W.); lujx@ninh.chinacdc.cn (J.L.); huyc@ninh.chinacdc.cn (Y.H.); limin@ninh.chinacdc.cn (M.L.); chenjing@ninh.chinacdc.cn (J.C.)

* Correspondence: yanglc@ninh.chinacdc.cn (L.Y.); maodq@ninh.chinacdc.cn (D.M.)

Supplementary materials and methods

Nutrients Components Analysis

1. Determination of Main Nutrients

The protein content of maize was determined using the Kjeldahl method. The solid sample, after digestion with concentrated sulfuric acid at 420 ℃ for 1 hour, was analyzed by the automated Kjeldahl nitrogen analyzer. The fat content was determined by acid hydrolysis, in which the sample was hydrolyzed with a hydrochloric acid solution to release bound lipids (70-80 ℃, water bath for 50 minutes), followed by extraction with ethyl ether and petroleum ether to determine the total fat content. The ash content of maize was determined by weighing the residue after combustion in a muffle furnace (900 ℃). The water content was determined by the oven-drying method. The dietary fiber content was determined using the enzymatic–gravimetric method. After enzymatic degradation to remove proteins and starches, the residue of the sample was subjected to ethanol precipitation, filtration, drying, and weighing. Additionally, the carbohydrate content was calculated by subtracting the contents of water, protein, fat, ash, and dietary fiber from the total sample weight.

2. Determination of Minerals

The calcium, potassium, phosphorus, magnesium, manganese, iron, copper, zinc, and sodium in the maize samples were determined by Inductively Coupled Plasma–Optical Emission Spectrometry (ICP-OES). After the samples were crushed and homogenized, nitric acid was added for microwave digestion. The instrument conditions were set as follows: power 1150 W; plasma gas flow 15 L/min; auxiliary gas flow 0.5 L/min; nebulizer gas flow 0.65 L/min; sample pump rate 50 rpm; and axial observation mode. Quantification was performed using the standard curve.

The selenium in the maize samples was determined by Hydride Generation Atomic Fluorescence Spectrometry. The samples were crushed and microwave digested. The conditions of the atomic fluorescence spectrometer were set as follows: negative high voltage 340 V; lamp current 100 mA; atomizer temperature 800 ℃; atomizer height 8 mm; carrier gas flow rate 500 mL/min; and shield gas flow rate 1000 mL/min. Quantification was performed using a standard curve method.

3. Determination of Vitamins

Vitamin A, δ-tocopherol, γ-tocopherol, β-tocopherol, and α-tocopherol in the maize samples were determined by reversed-phase high-performance liquid chromatography. The sample was analyzed after saponification and extraction. The chromatographic separation was performed on a C30 column (4.6 × 250 mm, 3 μm) using a mobile phase of methanol–water (96:4, v/v) at a flow rate of 0.8 mL/min. The column temperature was 20 °C, and detection wavelengths were set at 325 nm for vitamin A and 294 nm for vitamin E.

Vitamin K1 was determined by high-performance liquid chromatography–fluorescence detection. The sample was pretreated by enzymatic hydrolysis (37 °C, water bath for 2 hours) and n-hexane extraction.

Chromatographic separation was carried out on a C18 column (4.6 × 250 mm, 5 μm) using a mobile phase according to the standard formulation at a flow rate of 1 mL/min. Detection was performed at excitation and emission wavelengths of 243 nm and 430 nm, respectively, with post-column zinc reduction.

β-carotene was determined by high-performance liquid chromatography. The sample was analyzed after saponification and extraction. The chromatographic separation was achieved on a C18 column (4.6 × 250 mm, 5 μm) using a mobile phase of chloroform–acetonitrile–methanol (3:12:85, v/v/v) at a flow rate of 2.0 mL/min. The column temperature was 35°C, and detection was performed at 450 nm.

Vitamin B1 and B2 were determined by high-performance liquid chromatography. The sample was analyzed after extraction with an acidic buffer and enzymatic hydrolysis (37 °C, water bath for 16 hours). The chromatographic separation was performed on a C18 column (4.6 × 250 mm, 5 μm) using a mobile phase of 0.05 mol/L sodium acetate solution-methanol (65:35, v/v). The flow rate for vitamin B1 and B2 was 0.8 mL/min and 1.0 mL/min, respectively. The contents of niacin, folate and biotin in maize were determined by microbiological assay after acid hydrolysis with sulfuric acid solution. Lactobacillus plantarum ATCC 8014 was used as the indicator microorganism for niacin and biotin. Lactobacillus casei spp. rhamnosus ATCC 7469 was used for folate. The microorganisms were inoculated into the culture medium containing a series of dilutions of the test vitamin reference standards. The growth of microorganisms was determined by spectrophotometry, and a standard curve was constructed by plotting the correlation between absorbance and standard concentration. Subsequently, the vitamin content in samples was quantified using the interpolation method based on the sample absorbance values on this standard curve. The detection wavelengths are 550 nm for niacin and biotin, and 540 nm for folate. The folate analysis was performed in the dark.

Grouping and Dosage Information of gavage groups

All the rats in gavage groups were given AIN-93M ordinary diet. According to the effective dose reported in the literature [1], rats in the high-dose gavage (HDG) group were given astaxanthin equivalent to 0.02% of the dietary intake (20 mg astaxanthin per 100 g feed gavage). Rats in the medium-dose gavage (MDG) group were given 0.2 mg astaxanthin per 100 g feed gavage, which was 30 times the recommended daily dose of astaxanthin health product (6 mg/day). Rats in the low-dose gavage (LDG) group were given 0.001mg astaxanthin per 100g feed gavage. Feed for each group is shown in **Table S1**. The gavage groups were compared with the conventional feed group.

Table S1. Grouping and feeding of rats.

Group	Treatment group	Sample size	Feeding
Control group	Conventional feed (CF) group	10	AIN-93M ordinary diet
Experimental groups	High-dose gavage (HDG) group	16	AIN-93M ordinary diet and 20mg astaxanthin per 100g feed gavage
	Medium-dose gavage (MDG) group	10	AIN-93M ordinary diet and 0.2mg astaxanthin per 100g feed gavage
	Low-dose gavage (LDG) group	10	AIN-93M ordinary diet and 0.001mg astaxanthin per 100g feed gavage

Supplementary results

Table S2. Body weight changes and the comparison among astaxanthin-rich maize groups and control groups (g, Mean ± SD) [a].

Week	CF group	PM group	HDAM group	LDAM group
Week 0	693.09±70.03	691.77±80.80	691.20±73.95	689.83±75.49
Week 1	703.65±69.84	709.14±86.88	710.71±68.55	706.17±77.62
Week 2	725.13±70.60	734.64±93.02	735.64±68.76	725.29±84.61
Week 3	739.86±74.68	761.67±101.74	765.19±76.32	733.88±90.59
Week 4	746.61±77.79	775.73±109.51	777.18±82.22	752.69±94.00
Week 5	746.79±85.14	800.74±109.10	786.71±76.19	756.61±66.77
Week 6	755.85±87.75	815.70±116.72	802.02±76.50	777.33±68.86
Week 7	766.72±86.11	824.47±115.31	815.40±81.37	790.66±67.02
Week 8	772.90±84.95	837.03±116.89	825.01±79.52	806.34±70.03

[a] SD: standard deviation; CF: conventional feed; PM: parental maize; HDAM: high-dose astaxanthin-rich maize; LDAM: low-dose astaxanthin-rich maize.

Table S3. Food intake changes and the comparison among astaxanthin-rich maize groups and control groups (g/kg, Mean ± SD) [b].

Week	CF group	PM group	HDAM group	LDAM group
Week 1	45.47±4.57	48.11±8.27	48.54±6.31	46.14±4.77
Week 2	46.71±2.31	50.10±3.28	50.18±5.03 [a]	46.55±5.64
Week 3	48.24±3.12	48.20±1.20	49.28±4.53	44.71±3.68
Week 4	46.67 ±3.09	47.43±1.65	44.75±3.60	45.34±2.43
Week 5	46.88±2.81	42.36±4.20	43.70±2.78	44.43±3.17
Week 6	47.51±4.37	39.94±3.80	42.39±1.82 [a]	42.95±2.48
Week 7	48.93±3.59	41.20±1.65	44.96±1.96	44.15±2.45
Week 8	40.47±2.88	34.71±1.30	35.09±1.35 [a]	36.06±2.30

[a] Compared with the conventional feed group, $P < 0.05$. [b] SD: standard deviation; CF: conventional feed; PM: parental maize; HDAM: high-dose astaxanthin-rich maize; LDAM: low-dose astaxanthin-rich maize.

Table S4. The results of the repeated-measures analysis of variance [a].

Variables	Effects	F	df	*P*	η^2
Body weight	Time	12.711	1.717	<0.001	0.232
	Time×group	4.631	5.152	0.001	0.249
Food intake	Time	24.472	3.670	<0.001	0.550
	Time×group	2.024	11.009	0.038	0.233

a Mauchly's test indicated a violation of sphericity ($P < 0.05$). Greenhouse–Geisser corrected results are reported.

Table S5. Body weight changes and the comparison among gavage groups and control group (g, Mean ± SD) [b].

Week	CF group	HDG group	MDG group	LDG group
Week 0	693.09±70.03	689.41±51.35	690.72±64.30	693.96±63.26
Week 1	703.65±69.84	688.17±52.76	690.82±61.04	692.83±63.21
Week 2	725.13±70.60	684.57±52.98	694.28±56.57	702.43±62.49
Week 3	739.86±74.68	687.39±62.97	692.74±59.55	708.57±70.97
Week 4	746.61±77.79	683.51±72.95	697.77±69.37	703.38±85.90
Week 5	746.79±85.14	679.16±73.14 [a]	700.91±78.28	732.93±68.33
Week 6	755.85±87.75	680.51±80.16 [a]	708.76±71.32	736.22±69.42

Week 7	766.72±86.11	681.13±86.94 [a]	710.33±69.59	740.52±69.64
Week 8	772.90±84.95	684.14±87.53 [a]	716.03±70.75	734.96±76.76

[a] Compared with the conventional feed group, $P < 0.05$. [b] SD: standard deviation; CF: conventional feed; HDG: high-dose gavage; MDG: medium-dose gavage; LDG: low-dose gavage.

Table S6. Food intake changes and the comparison among gavage groups and control group (g/kg, Mean ± SD) [b].

Week	CF group	HDG group	MDG group	LDG group
Week 1	45.47±4.57	41.00±4.22	45.68±3.55	44.96±2.89
Week 2	46.71±2.31	38.13±2.85 [a]	40.05±4.21 [a]	43.76±2.42
Week 3	48.24±3.12	42.46±1.62 [a]	43.57±1.34	47.94±2.04
Week 4	46.67 ±3.09	41.00±4.71 [a]	44.95±3.15	45.62±2.38
Week 5	46.88±2.81	44.21±1.93	46.44±7.58	44.78±1.65
Week 6	47.51±4.37	43.28±2.38 [a]	42.67±2.68 [a]	43.69±2.34
Week 7	48.93±3.59	43.45±4.23 [a]	42.16±4.89 [a]	45.22±2.17
Week 8	40.47±2.88	36.55±3.54	36.46±2.51	34.40±6.59 [a]

[a] Compared with the conventional feed group, $P < 0.05$. [b] SD: standard deviation; CF: conventional feed; HDG: high-dose gavage; MDG: medium-dose gavage; LDG: low-dose gavage.

Table S7. Effect of astaxanthin-rich maize feeding experiment on serum oxidative stress indicators of rats (Mean ± SD) [e].

Group	CF group (n=10)	HDG group (n=16)	MDG group (n=9)	LDG group (n=9)	F	df	*P*
Protein carbonyl (nmol/ml)	0.68±0.22	0.65±0.32	0.95±0.68	1.29±0.56 [ab]	3	4.589	0.007
SOD (U/ml)	366.83±26.16	403.12±19.38 [a]	435.23±34.65 [ab]	399.12±36.64 [c]	3	9.758	<0.001
MDA (nmol/ml) [d]	15.45±4.06	8.31±3.24 [a]	6.15±0.89 [a]	10.50±1.31 [ac]	3	34.105	<0.001
GSH (mg/L)	68.84±13.35	118.24±18.37 [a]	148.14±10.15 [ab]	79.32±8.20 [bc]	3	60.894	<0.001
GPX (U/ml) [d]	925.94±213.68	1179.88±320.07	806.04±213.41 [b]	1029.97±189.16	3	4.251	0.017
8-isoprostane (ng/L)	63.80±43.97	54.34±23.47	55.94±34.78	107.00±46.01 [bc]	3	4.578	0.007

[a] Compared with the CF group, $P < 0.05$. [b] Compared with the HGD group, $P < 0.05$. [c] Compared with the MDG group, $P < 0.05$. [d] Data for MDA and GPX were analyzed using Welch's ANOVA due to heterogeneity of variances. [e] SD: standard deviation; SOD: superoxide dismutase; MDA: malondialdehyde; GSH: glutathione; GPX: glutathione peroxidase; CF: conventional feed; HDG: high-dose gavage; MDG: medium-dose gavage; LDG: low-dose gavage.

Table S8. Effect of astaxanthin-rich maize feeding experiment on liver oxidative stress indicators of rats (Mean ± SD) [d].

Group	CF group (n=10)	HDG group (n=16)	MDG group (n=9)	LDG group (n=9)	F	df	*P*
Protein carbonyl (nmol/ml) [c]	10.24± 4.46	4.20±2.30 [a]	3.71±1.79 [a]	3.73±1.52 [a]	5.808	3	0.009
SOD (U/ml)	349.88±44.83	322.51±34.98	347.66±28.46	324.95±57.82	1.348	3	0.273
MDA (nmol/ml)	1.07±0.22	1.02±0.23	0.92±0.16	1.13±0.15	1.764	3	0.170
GSH (mg/L) [c]	1.49±0.53	0.88±0.50 [a]	0.71±0.39 [a]	1.57±0.89	5.665	3	0.006
GPX (U/ml) [c]	539.18±86.58	361.23±93.95 [a]	405.56±173.35	565.93±98.91 [b]	10.865	3	<0.001
8-isoprostane (ng/L)	92.19±17.57	97.73±13.15	95.46±18.58	85.68±18.90	1.079	3	0.369

[a] Compared with the CF group, $P < 0.05$. [b] Compared with the HGD group, $P < 0.05$. [c] Data for protein carbonyl, GSH, and GPX were analyzed using Welch's ANOVA due to heterogeneity of variances. [d] SD: standard deviation; SOD: superoxide dismutase; MDA: malondialdehyde; GSH: glutathione; GPX: glutathione peroxidase; CF: conventional feed; HDG: high-dose gavage; MDG: medium-dose gavage; LDG: low-dose gavage.

Reference

1. Naito, Y.; Uchiyama, K.; Aoi, W.; Hasegawa, G.; Nakamura, N.; Yoshida, N.; Maoka, T.; Takahashi, J.; Yoshikawa, T., Prevention of diabetic nephropathy by treatment with astaxanthin in diabetic db/db mice. *BioFactors (Oxford, England)* **2004,** *20*, 49-59.
